# Association of type 2 diabetes with family history of diabetes, diabetes biomarkers, mental and physical disorders in a Kenyan setting

**DOI:** 10.1038/s41598-024-61984-6

**Published:** 2024-05-14

**Authors:** David M. Ndetei, Victoria Mutiso, Christine Musyimi, Pascalyne Nyamai, Cathy Lloyd, Norman Sartorius

**Affiliations:** 1 Department of Psychiatry, University of Nairobi, Nairobi, Kenya; 2 Africa Mental Health Research and Training Foundation, Mawensi Road, Off Elgon Road, Mawensi Garden, P.O. Box 48423-00100, Nairobi, Kenya; 3 World Psychiatric Association Collaborating Centre for Research and Training, Nairobi, Kenya; 4 The Open University, Milton Keynes, UK; 5 Association for the Improvement of Mental Health Programmes (AMH), Geneva, Switzerland

**Keywords:** Family relations, Type 2 diabetes mellitus, Depression, Physical conditions, Kenyan setting, Psychology, Endocrinology, Risk factors

## Abstract

This study aimed to determine the degree of family relations and associated socio-demographics characteristics, clinical/physical and mental disorders in type 2 diabetes mellitus in a Kenyan diabetes clinic. This study was part of a large multicentre study whose protocol and results had been published. It took place at the outpatient diabetes clinic at a County Teaching and Referral Hospital in South East Kenya involving 182 participants. We used a socio-demographic questionnaire, the Hamilton Depression (HAM-D) and PHQ-9 rating scales for depression, the MINI International Neuropsychiatric Interview (MINI; V5 or V6) for DSM-5 diagnoses, the WHO-5 Well-being scale and Problem Areas in Diabetes Scale (PAID). We extracted from the notes all physical conditions. We enquired about similar conditions in 1st and 2nd degree relatives. Descriptive, Chi-square test, Fisher’s exact test, one way ANOVA, and Multinomial logistic regression analysis were conducted to test achievements of our specific aims. Of the 182 patients who participated in the study, 45.1% (82/182) reported a family history of diabetes. Conditions significantly (*p* < 0.05) associated with a degree of family history of diabetes were retinopathy, duration of diabetes (years), hypertension, and depressive disorder. On average 11.5% (21/182) scored severe depression (≥ 10) on PHQ-9 and 85.2% (115/182) scored good well-being (≥ 13 points). All DSM-5 psychiatric conditions were found in the 182 patients in varying prevalence regardless of relations. In addition, amongst the 182 patients, the highest prevalence was poor well-being on the WHO quality of life tool. This was followed by post-traumatic disorders (current), suicidality, and psychotic lifetime on DSM-5. The least prevalent on DSM-5 was eating disorders. Some type 2 diabetes mellitus physical disorders and depression have increased incidence in closely related patients. Overall, for all the patients, the prevalence of all DSM-5 diagnoses varied from 0.5 to 9.9%.

## Introduction

Family history is a non-modifiable risk factor for diabetes^1–8^. The risk of developing type 2 diabetes mellitus (T2DM) increases approximately two to four times when either or both parents have T2DM^5^. Between 60 and 68.8% of diabetes patients have at least one family member with a history of diabetes^2,6^. Paternal history is significantly associated with higher chances of having T2DM^1^. An early age onset of T2DM is more likely if a family member had also an early onset of diabetes^2,8–10^.

A positive family history of diabetes is associated with increased levels of obesity, impaired glucose tolerance, fasting triglycerides, hemoglobin AIc (HbA1c), insulin dose per kilogram, lower levels of high-density lipoprotein cholesterol^3,8,11,12^, a greater waist to hip ratio as well as greater body mass index (BMI)^13^ and a high prevalence of diabetes complications, particularly retinopathy and dyslipidemia compared to those without a relative with diabetes^9^. More specifically and in addition, there is an impact on leptin, (a hormone that regulates fat storage in the body)^14^, a high prevalence of hypertension, and lower low-density lipoprotein (LDL) cholesterol levels in those with fathers with T2DM as opposed to those with mothers with diabetes^15^. Various physical conditions are associated with diabetes. These include cardiovascular diseases^16,17^, hypertension^18^, thyroid abnormality, and diabetes complications such as retinopathy, neuropathy, and stroke^17,19,20^ as well as high levels of biomarkers such as hemoglobin AIc (HbAIc) and cholesterol^21^.

Mental disorders such as schizophrenia, major depressive disorder, and bipolar disorder are associated with a family history of diabetes^22–24^. The risk of developing diabetes is three times higher in individuals with schizophrenia than in the normal population^23^. Siblings of schizophrenic parents are more likely to develop T2DM than those whose parents do not have schizophrenia^25^.

T2DM is also associated with anxiety, Post Traumatic Stress Disorder (PTSD), depression^26–28^, and eating disorders^29–31^. Research in this area is lacking in a Kenyan setting and is urgently required in order to inform clinical practice and potential community-based interventions.

Studies in African countries on the association between diabetes and family history have largely confirmed the global trends, showing an increased frequency of T2DM in persons with a family history of diabetes and an early onset of diabetes between 18 and 30 years^32–35^. Significantly higher blood glucose levels have been reported in those with a maternal family history of diabetes than in those without such a history^36^. Kenyan studies have found that people with T2DM are likely to have a positive family history specifically a first-degree relative and are also likely to develop diabetes early in life^37,38^. First-degree relations include an individual's biological parents, siblings, and children. Second-degree relatives include an individual's grandparents, grandchildren, uncles, aunts, nephews, nieces, and half-siblings. No study in Kenya has examined how physical conditions and mental disorders are comorbid in patients with T2DM or has examined the degree of family relations and how these vary with socio-demographics, measures of well-being, stress levels related to diabetes, and the prevalence of DSM-5 diagnoses in the Type 2 Diabetes. This information would inform an integrated approach to management. This study sought to fill these gaps.

The primary objective of this study was to determine the degree of family relations and associated socio-demographic characteristics, physical and mental disorders in people with T2DM. The secondary objective was to determine the overall prevalences of physical disorders and mental disorders in T2DM regardless of family relations.

The primary specific aims were:

1. To determine the relationships between social demographics in T2DM in different degrees of family relations
2. To determine the patterns of physical disorders and physical characteristics of T2DM in different degrees of family relations
3. To determine the mental health and disorders associated with T2DM in different degrees of family relations
4. To determine the independent predictors of T2DM in different degrees of family relations

The secondary specific aims were:

1. To determine the overall prevalence of physical disorders in T2DM
2. To determine the overall prevalence of mental disorders (stress, wellbeing, and psychiatric disorders) in T2DM
3. To determine the independent predictors of depression in T2DM

## Methods

### Study design and setting

This study was part of a larger multicentre study whose protocol has been published previously^39^. It took place between September 2013 and May 2015 at an outpatient diabetes clinic in one of the County Teaching and Referral Hospital in South East Kenya approximately 60 Kilometres from Nairobi. The clinic is run by a diabetologist and a team trained in diabetes management, offering psychoeducation, and counselling.

### Study participants

Between September 2013 and May 2015, a sample of consecutive outpatient clinic attendees with T2DM were invited to participate in the study. Inclusion criteria were adults aged 18–65 with T2DM diagnosed at least 12 months earlier and able to give informed consent. Exclusion criteria included: communication and cognitive difficulties; life threatening or serious conditions in the previous 6 months and being an inpatient (as this may have indicated a serious condition); pregnant women or in the first 6 months post-partum clinic; substance use dependency or a current schizophrenic illness. All patients who met the inclusion criteria and did not have any exclusion criteria consented to the study and were included.

The trained research assistant completed a form that contained information from the medical records such as age, duration of diabetes, and presence/history of diabetes complications i.e. cardiovascular disease, retinopathy, peripheral neuropathy, peripheral vascular disease, and renal disease and associated disorders as well as the most recent measurement of blood pressure, HbA1c, height and weight.

For this study, we recorded the family history of T2DM in the following:

i. History of diabetes in 1st degree relatives (parent or sibling)
ii. History of diabetes in 2nd degree relatives (grandparents, aunt, uncle, and cousin)
iii. History of diabetes in both 1st and 2nd degree relatives

### Study instruments

- A standardised template for extracting information from the medical records on socio-demographic data and various medical complications known to be associated with T2DM, and laboratory indicators of T2DM was utilised. We also enquired about the history of smoking.
- The following psychometric instruments were administered by a trained research assistant: (i) the Patient Health Questionnaire (PHQ-9), (ii) the Hamilton Depression (HAM-D) rating scale, (iii) the WHO-5 wellbeing scale, (iv) the Problem Areas in Diabetes Scale (PAID) and (v) the MINI International Neuropsychiatric Interview (MINI; V5 or V6). The psychometric properties of these instruments have been described in the protocol for this study^39^ but also summarized here for quick reference. The PHQ-9 consists of 9 items on a 4-point Likert-type scale (0 = not at all; 1 = several days; 2 = more than half the days; 3 = nearly every day) with a total score ranging from 0–27. It has good psychometric properties and has been used extensively in many culturally diverse countries^40^. PHQ-9 scores with cut-off points of 1, 5, 10, 15, and 20 represent minimal, mild, moderate, moderately severe, and severe depression, respectively. Moderate to severe depressive symptomatology was defined as PHQ-9 scores > 10, as this was a research study rather than clinical practice where a significant level of symptoms would usually be considered as PHQ-9 scores above 15^41^. The Hamilton Depression (HAM-D) Rating Scale has been considered a gold standard in depression studies and a preferred scale in the evaluation of depression treatment^42^.

It is the most widely employed depression scale on a global scale^43^ and has been administered to various patient populations ranging from psychiatric, medical, and other research settings^44^. The HAM-D Rating Scale is a 17-item tool that takes 20–30 min to administer and scored between 0 and 4 points. Scores of 0–7 indicate normal, 8–16 indicate mild depression, 17–23 moderate depression, and counts over 24 are indicative of severe depression^42^. It has good psychometric properties with sufficient reliability (internal, inter-rater, and retest safety) and efficacy (convergent, discriminant, and predictive validity)^44^. The WHO-5 wellbeing scale is a 5-item questionnaire that measures a person’s overall psychological wellbeing^45^. The items are ‘I have felt cheerful and in good spirits’, ‘I have felt calm and relaxed’, ‘I have felt active and vigorous’, ‘I woke up feeling fresh and rested’, and ‘My daily life has been filled with things that interest me’. Poor wellbeing was defined as WHO-5 scores < 13. The PAID is a 20-item questionnaire which measures the extent of diabetes-related emotional distress^46^. Items include ‘feeling overwhelmed with your diabetes’ and ‘feelings of guilt or anxiety when you get off track with your diabetes management’. Moderate-severe levels of diabetes-related distress are defined as scores (standardized to 100) > 40^46^. The MINI has been widely used in a range of different populations—including those with serious illnesses and in community surveys and is a reliable diagnostic tool according to DSM-V criteria^47^. It can be administrated by trained non-mental health specialists. Individuals diagnosed with depression (or other psychiatric disorders such as anxiety disorders) were advised to consult their physician for further assessment and treatment with a view to referral to the hospital psychiatric services. If any individual indicated suicidality (question 9 on the PHQ-9) immediate referral was made to the psychiatric service at the hospital.

### Ethical consideration

Ethical approval was granted by the Kenyatta National Hospital—University of Nairobi (KNH-UoN) Ethics and Research Committee (ERC) (#P470/09/2013). All methods were performed in accordance with relevant guidelines and regulations as per the World Medical Association Declaration of Helsinki—ethical principles for medical research involving human subjects. Informed written consent was obtained from participants. For illiterate participants, informed written consent was obtained from their guardian/legally authorised representative.

### Data analysis

This was performed using SPSS version 21 (IBM, Chicago, IL). All continuous variables were tested for normality using the Shapiro–Wilk test. Basic descriptive statistics in the form of frequency, percentage, mean, and standard deviation were carried out. The chi-square test or Fisher's exact test were used where appropriate to analyze the difference in the prevalence between family history of diabetes across different categories of socio-demographics, physical and mental disorder variables. Differences in levels of continuous variables were examined using the one way ANOVA for parametric data. Multinomial logistic regression was employed to identify the impact of a family history of diabetes on the risk factors of diabetes in the participants. Statistical significance was considered at *p* value < 0.05.

## Results

### Socio-demographic characteristics

Table 1 summarizes the socio-demographic characteristics (frequencies and percentages) of the participants and the association between the degree of family history of diabetes and socio-demographic characteristics.

**Table 1:** Socio-demographics, smoking status and family relations in T2DM.

Variable	Category	Total N = 182	Family history of diabetes*				
			No family history	Diabetes in 1st degree relative (parent, sibling)	Diabetes in 2nd degree relative (grandparent, aunt, uncle, cousin)	Diabetes in both 1st and 2nd degree relatives	*p* value
Gender	Female	135 (74.2%)	67 (67.0%)	37 (84.1%)	17 (77.3%)	14 (87.5%)	0.098^†^
	Male	47 (25.8%)	33 (33.0%)	7 (15.9%)	5 (22.7%)	2 (12.5%)	
Level of education status	No formal education	8 (4.40%)	3 (3.00%)	3 (6.82%)	0 (0%)	2 (12.5%)	0.074^†^
	Some/completed primary school	82 (45.1%)	54 (54.0%)	13 (29.5%)	10 (45.5%)	5 (31.2%)	
	Some/completed secondary school	76 (41.8%)	37 (37.0%)	21 (47.7%)	11 (50.0%)	7 (43.8%)	
	Higher education (college, post-grad/professional)	16 (8.79%)	6 (6.00%)	7 (15.9%)	1 (4.55%)	2 (12.5%)	
Marital status	Married/co-habiting	143 (78.6%)	78 (78.0%)	34 (77.3%)	18 (81.8%)	13 (81.2%)	0.468^†^
	Single	15 (8.24%)	10 (10.0%)	2 (4.55%)	3 (13.6%)	0 (0%)	
	Widowed	21 (11.5%)	9 (9.00%)	8 (18.2%)	1 (4.55%)	3 (18.8%)	
	Divorced	3 (1.65%)	3 (3.00%)	0 (0%)	0 (0%)	0 (0%)	
Family income status	No regular income (e.g. unemployed/student)	61 (33.7%)	37 (37.4%)	16 (36.4%)	6 (27.3%)	2 (12.5%)	0.225^‡^
	Regular income (e.g. part/full-time work, pension)	120 (66.3%)	62 (62.6%)	28 (63.6%)	16 (72.7%)	14 (87.5%)	
Location of residence	Rural/Village	149 (81.9%)	77 (77.0%)	37 (84.1%)	21 (95.5%)	14 (87.5%)	0.209^†^
	Urban	33 (18.1%)	23 (23.0%)	7 (15.9%)	1 (4.55%)	2 (12.5%)	
Availability of health services in location of residence	No	18 (9.89%)	11 (11.0%)	4 (9.09%)	2 (9.09%)	1 (6.25%)	> 0.999^†^
	Yes	164 (90.1%)	89 (89.0%)	40 (90.9%)	20 (90.9%)	15 (93.8%)	
Exercise frequency	Rarely/never/monthly	46 (25.4%)	28 (28.0%)	12 (27.3%)	5 (23.8%)	1 (6.25%)	0.310^†^
	Daily/weekly	135 (74.6%)	72 (72.0%)	32 (72.7%)	16 (76.2%)	15 (93.8%)	
Age group	50 and below	80 (44.0%)	46 (46.0%)	20 (45.5%)	11 (50.0%)	3 (18.8%)	0.337^†^
	51–60	72 (39.6%)	35 (35.0%)	19 (43.2%)	8 (36.4%)	10 (62.5%)	
	Over 60	30 (16.5%)	19 (19.0%)	5 (11.4%)	3 (13.6%)	3 (18.8%)	
Smoking status	Never	165 (90.7%)	88 (88.0%)	41 (93.2%)	20 (90.9%)	16 (100.0%)	0.643^†^
	Past	15 (8.24%)	11 (11.0%)	2 (4.55%)	2 (9.09%)	0 (0%)	
	Current	2 (1.10%)	1 (1.00%)	1 (2.27%)	0 (0%)	0 (0%)	

*Column percentages.

^‡^Chi-square test.

^†^Fisher’s exact test; *p* value = significance level.

The mean age was 50.1 (± 11.1) years. The majority of respondents were female (74.2%), married/co-habiting (78.6%), had a regular income household (66.3%), were daily/weekly exercisers (74.6%) and non-smokers (90.7%), with the smallest proportion living in an urban area (18.1%) and the biggest proportion having access to health services (90.1%).

Of the 182 study participants, 45.1% (82/182) reported a family history of diabetes. The prevalence of diabetes in 1st degree relatives (parent, sibling) and 2nd degree relatives (grandparent, aunt, uncle, cousin) was 24.2% (44/182) and 12.1% (22/182) respectively; 8.8% (16/182) reported a family history of diabetes in both 1st degree and 2nd degree relatives.

The degree of family history of diabetes was not significantly (*p* > 0.05) associated with any socio-demographic variable.

### Physical conditions and clinical characteristics in family relations

**Figure 1:**
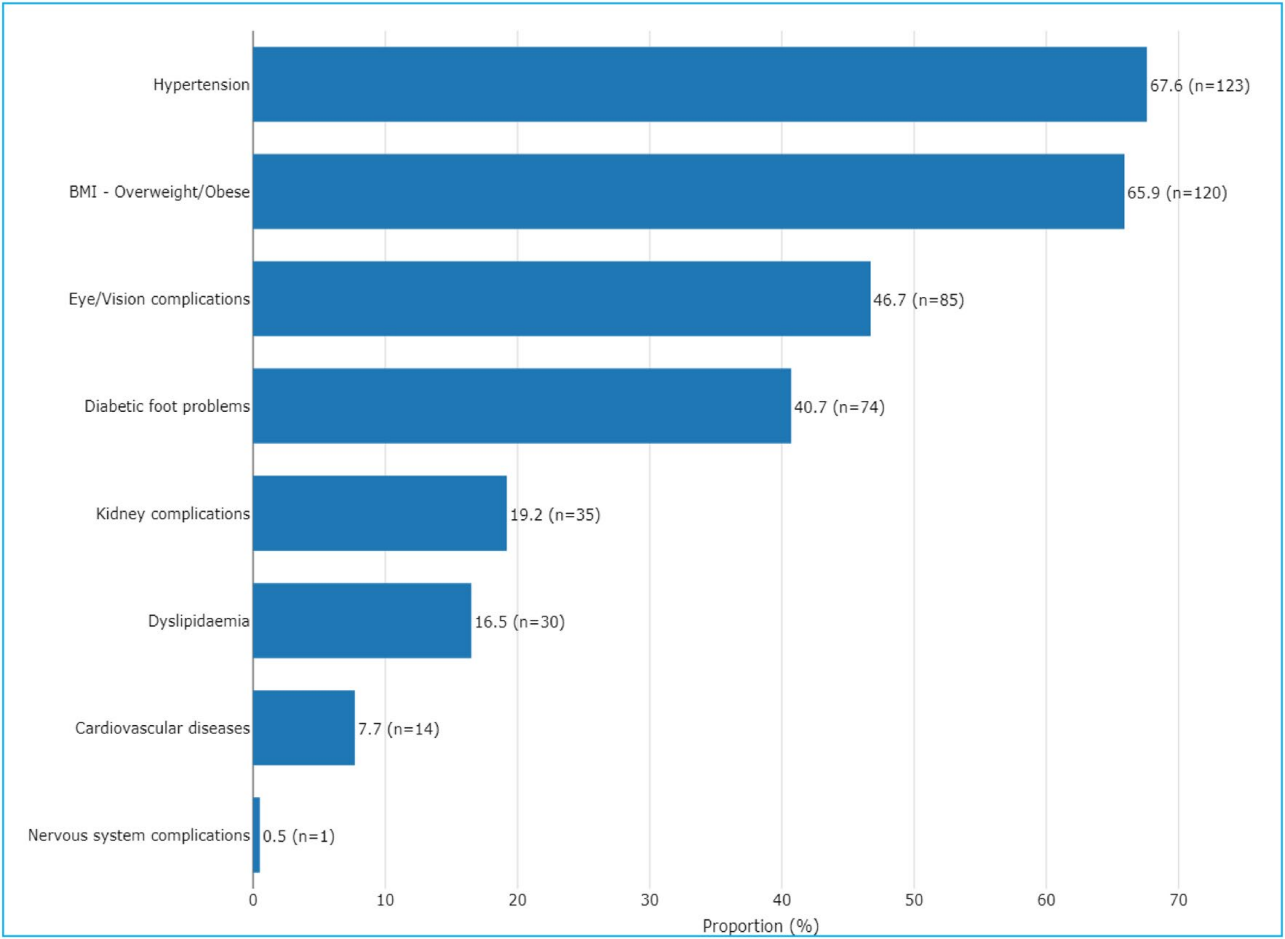
Prevalence of the various physical conditions in descending order (N = 182).

Table 2 summarizes the associations between the degree of family history of diabetes and physical conditions/clinical characteristics while Fig. 1 summarizes various physical conditions in descending prevalence.

**Table 2:** Physical conditions and family relations in T2DM.

Variable	Category	Total N = 182	Family history of diabetes*				
			No family history	Diabetes in 1st degree relative (parent, sibling)	Diabetes in 2nd degree relative (grandparent, aunt, uncle, cousin)	Diabetes in both 1st and 2nd degree relatives	*p* value
Physical conditions							
Retinopathy	No	123 (72.8%)	70 (76.1%)	32 (74.4%)	17 (85.0%)	4 (28.6%)	**0.003**^**†**^
	Yes	46 (27.2%)	22 (23.9%)	11 (25.6%)	3 (15.0%)	10 (71.4%)	
Duration of diabetes (years)	Mean (SD)	7.21 (6.11)	6.85 (6.01)	7.30 (5.93)	5.36 (4.05)	11.81 (7.70)	**0.009**^a^
Diabetes medications	No diabetes medications	2 (1.11%)	2 (2.04%)	0 (0%)	0 (0%)	0 (0%)	0.113^†^
	Oral hypoglycemic agents only	104 (57.8%)	51 (52.0%)	31 (70.5%)	11 (50.0%)	11 (68.8%)	
	Insulin only	58 (32.2%)	39 (39.8%)	7 (15.9%)	8 (36.4%)	4 (25.0%)	
	Both oral agents and insulin	16 (8.89%)	6 (6.12%)	6 (13.6%)	3 (13.6%)	1 (6.25%)	
BMI	Low/Normal	58 (32.6%)	32 (32.7%)	15 (34.1%)	7 (35.0%)	4 (25.0%)	0.915^‡^
	Overweight/Obese	120 (67.4%)	66 (67.3%)	29 (65.9%)	13 (65.0%)	12 (75.0%)	
Kidney complications	No	136 (79.5%)	71 (77.2%)	37 (84.1%)	18 (85.7%)	10 (71.4%)	0.611^†^
	Yes	35 (20.5%)	21 (22.8%)	7 (15.9%)	3 (14.3%)	4 (28.6%)	
Diabetic foot problems	No	100 (57.5%)	51 (53.1%)	27 (61.4%)	15 (75.0%)	7 (50.0%)	0.278^‡^
	Yes	74 (42.5%)	45 (46.9%)	17 (38.6%)	5 (25.0%)	7 (50.0%)	
Cardiovascular diseases	No	158 (91.9%)	85 (91.4%)	42 (95.5%)	20 (90.9%)	11 (84.6%)	0.523^†^
	Yes	14 (8.14%)	8 (8.60%)	2 (4.55%)	2 (9.09%)	2 (15.4%)	
Nervous system complications	No	168 (99.4%)	89 (98.9%)	44 (100.0%)	21 (100.0%)	14 (100.0%)	> 0.999^†^
	Yes	1 (0.59%)	1 (1.11%)	0 (0%)	0 (0%)	0 (0%)	
Hypertension	No	58 (32.0%)	31 (31.3%)	13 (29.5%)	12 (54.5%)	2 (12.5%)	**0.045**^**‡**^
	Yes	123 (68.0%)	68 (68.7%)	31 (70.5%)	10 (45.5%)	14 (87.5%)	
Dyslipidemia	No	148 (83.1%)	84 (85.7%)	33 (76.7%)	17 (81.0%)	14 (87.5%)	0.566^†^
	Yes	30 (16.9%)	14 (14.3%)	10 (23.3%)	4 (19.0%)	2 (12.5%)	
Biochemistry biomarkers							
HbA1C (%)	Mean (SD)	10.02 (4.21)	9.69 (4.15)	9.77 (3.69)	9.84 (4.45)	12.99 (4.73)	**0.031**^a^
BP (mmHg)	BP ≥ 120/80	140 (76.9%)	74 (74.0%)	39 (88.6%)	13 (59.1%)	14 (87.5%)	**0.032**^**‡**^
	BP < 120/80	42 (23.1%)	26 (26.0%)	5 (11.4%)	9 (40.9%)	2 (12.5%)	

Significant values are in [bold].

*Column percentages.

^a^One way anova test.

^‡^Chi-square test.

^†^Fisher’s exact test; *p* value = significance level; Kidney complications (Nephropathy or kidney problems); Diabetic foot problems (Peripheral vascular disease or feet/leg problems); Cardiovascular diseases (Stroke/cerebrovascular incident or Heart attack/myocardial infarction or angina or heart disease/heart problems); Nervous system complications (Neuropathy or Nervous system problems); Hypertension (blood pressure medication or high blood pressure); dyslipidemia (Cholesterol medication or high cholesterol); BP = Blood pressure.

The physical conditions significantly (*p* < 0.05) associated with the degree of family history of diabetes were retinopathy, duration of diabetes (years), and history of hypertension. The clinical characteristics significantly (*p* < 0.05) associated with the degree of family history of diabetes were HbA1C (%) and hypertension.

### Mental disorders

Table 3 summarizes the association between the degree of family history of diabetes and mental disorders, mean scores of HAM-D, WHO-5 Well-being, PAID, and PHQ-9. It also summarizes the various DSM-5 diagnoses.

**Table 3:** DSM-V diagnoses in family relations mental disorders in T2DM.

Variable	Category	Total N = 182	Family history of diabetes*				
			No family history	Diabetes in 1st degree relative (parent, sibling)	Diabetes in 2nd degree relative (grandparent, aunt, uncle, cousin)	Diabetes in both 1st and 2nd degree relatives	*p* value
HAM-D total score	Mean (SD)	5.91 (6.19)	6.15 (6.86)	7.23 (5.60)	4.95 (4.86)	2.06 (2.32)	**0.030**^a^
WHO-5 total score	Mean (SD)	19.02 (5.47)	18.38 (6.05)	19.11 (4.49)	20.45 (4.57)	20.75 (4.84)	0.213^a^
PAID total score	Mean (SD)	7.76 (10.28)	7.68 (10.43)	7.16 (9.23)	10.74 (14.13)	5.78 (3.90)	0.461^a^
PHQ-9	Mean (SD)	4.70 (4.15)	4.90 (4.11)	4.69 (3.72)	5.05 (5.88)	3.00 (2.19)	0.384^a^
Major depressive episode current	No	177 (97.3%)	96 (96.0%)	43 (97.7%)	22 (100.0%)	16 (100.0%)	> 0.999^†^
	Yes	5 (2.75%)	4 (4.00%)	1 (2.27%)	0 (0%)	0 (0%)	
Major depressive episode recurrent	No	167 (91.8%)	91 (91.0%)	41 (93.2%)	20 (90.9%)	15 (93.8%)	0.972^†^
	Yes	15 (8.24%)	9 (9.00%)	3 (6.82%)	2 (9.09%)	1 (6.25%)	
Major depressive episode—with melancholic features	No	171 (94.0%)	93 (93.0%)	41 (93.2%)	21 (95.5%)	16 (100.0%)	0.919^†^
	Yes	11 (6.04%)	7 (7.00%)	3 (6.82%)	1 (4.55%)	0 (0%)	
Dysthymia current	No	171 (94.0%)	95 (95.0%)	41 (93.2%)	19 (86.4%)	16 (100.0%)	0.335^†^
	Yes	11 (6.04%)	5 (5.00%)	3 (6.82%)	3 (13.6%)	0 (0%)	
Suicidality current	No	165 (90.7%)	93 (93.0%)	38 (86.4%)	19 (86.4%)	15 (93.8%)	0.457^†^
	Yes	17 (9.34%)	7 (7.00%)	6 (13.6%)	3 (13.6%)	1 (6.25%)	
Hypomanic episode current	No	174 (95.6%)	95 (95.0%)	43 (97.7%)	21 (95.5%)	15 (93.8%)	0.888^†^
	Yes	8 (4.40%)	5 (5.00%)	1 (2.27%)	1 (4.55%)	1 (6.25%)	
Bipolar I disorder current	No	180 (98.9%)	99 (99.0%)	43 (97.7%)	22 (100.0%)	16 (100.0%)	0.699^†^
	Yes	2 (1.10%)	1 (1.00%)	1 (2.27%)	0 (0%)	0 (0%)	
Panic disorder current	No	174 (95.6%)	95 (95.0%)	43 (97.7%)	21 (95.5%)	15 (93.8%)	0.888^†^
	Yes	8 (4.40%)	5 (5.00%)	1 (2.27%)	1 (4.55%)	1 (6.25%)	
Panic disorder lifetime	No	165 (90.7%)	91 (91.0%)	38 (86.4%)	21 (95.5%)	15 (93.8%)	0.711^†^
	Yes	17 (9.34%)	9 (9.00%)	6 (13.6%)	1 (4.55%)	1 (6.25%)	
Agoraphobia current	No	172 (94.5%)	94 (94.0%)	42 (95.5%)	22 (100.0%)	14 (87.5%)	0.386^†^
	Yes	10 (5.49%)	6 (6.00%)	2 (4.55%)	0 (0%)	2 (12.5%)	
Social phobia (social anxiety disorder) current	No	171 (94.0%)	91 (91.0%)	42 (95.5%)	22 (100.0%)	16 (100.0%)	0.388^†^
	Yes	11 (6.04%)	9 (9.00%)	2 (4.55%)	0 (0%)	0 (0%)	
Obsessive–compulsive disorder current	No	176 (96.7%)	96 (96.0%)	42 (95.5%)	22 (100.0%)	16 (100.0%)	0.918^†^
	Yes	6 (3.30%)	4 (4.00%)	2 (4.55%)	0 (0%)	0 (0%)	
Post-traumatic stress disorder current	No	164 (90.1%)	88 (88.0%)	40 (90.9%)	20 (90.9%)	16 (100.0%)	0.632^†^
	Yes	18 (9.89%)	12 (12.0%)	4 (9.09%)	2 (9.09%)	0 (0%)	
Alcohol dependence	No	170 (93.4%)	91 (91.0%)	44 (100.0%)	20 (90.9%)	15 (93.8%)	0.129^†^
	Yes	12 (6.59%)	9 (9.00%)	0 (0%)	2 (9.09%)	1 (6.25%)	
Alcohol abuse	No	173 (95.1%)	94 (94.0%)	43 (97.7%)	20 (90.9%)	16 (100.0%)	0.531^†^
	Yes	9 (4.95%)	6 (6.00%)	1 (2.27%)	2 (9.09%)	0 (0%)	
Psychotic disorders lifetime	No	166 (91.2%)	90 (90.0%)	40 (90.9%)	20 (90.9%)	16 (100.0%)	0.753^†^
	Yes	16 (8.79%)	10 (10.0%)	4 (9.09%)	2 (9.09%)	0 (0%)	
Generalized anxiety disorder current	No	170 (93.4%)	92 (92.0%)	42 (95.5%)	21 (95.5%)	15 (93.8%)	0.961^†^
	Yes	12 (6.59%)	8 (8.00%)	2 (4.55%)	1 (4.55%)	1 (6.25%)	

Significant value is in [bold].

*Column percentages.

^a^One way anova test.

^†^Fisher’s exact test; *p* value, Significance level; HAM-D, Hamilton Depression Rating Scale 17 items; WHO-5, The World Health Organisation Well-Being Index 5 items; PAID, Problem Areas in Diabetes Questionnaire 20 items.

Only depressive symptoms (as measured by the HAM-D) were significantly (*p* = 0.030) associated with the degree of family history of diabetes. PHQ-9 unlike HAM-D did not reveal any significant trends (*p* > 0.05). All other measures were not significantly associated with a family history of diabetes (*p* > 0.05).

### Independent predictors of T2DM in family relations

Table 4 summarizes the predictors of T2DM in different degrees of family relations.

**Table 4:** Independent predictors of T2DM in different degrees of family relations.

Variable	Category	Diabetes in 1st degree relative (parent, sibling)		Diabetes in 2nd degree relative (grandparent, aunt, uncle, cousin)		Diabetes in both 1st and 2nd degree relatives	
		AOR (95% CI)	*p* value	AOR (95% CI)	*p* value	AOR (95% CI)	*p* value
Retinopathy	No	Ref		Ref		Ref	
	Yes	1.12 (0.48–2.65)	0.788	0.44 (0.11–1.76)	0.247	6.28 (1.36–28.88)	**0.018**
Duration of diabetes (years)	Mean (SD)	1.03 (0.97–1.11)	0.344	0.99 (0.88–1.10)	0.794	1.14 (1.02–1.27)	**0.023**
Hypertension	No	Ref		Ref		Ref	
	Yes	0.81 (0.34–1.90)	0.622	0.36 (0.12–1.05)	0.062	6.15 (0.55–68.31)	0.140
HbA1C (%)	Mean (SD)	1.02 (0.92–1.12)	0.757	1.00 (0.89–1.13)	0.976	1.21 (1.02–1.44)	**0.027**
BP (mmHg)	BP < 120/80	Ref		Ref		Ref	
	BP ≥ 120/80	2.91 (0.98–8.63)	0.054	0.53 (0.18–1.58)	0.253	2.13 (0.33–13.86)	0.429
HAM-D Total Score	Mean (SD)	1.01 (0.95–1.08)	0.662	0.98 (0.89–1.08)	0.621	0.67 (0.49–0.91)	**0.010**

Significant values are in [bold].

AOR, Adjusted odds ratio; CI, Confidence interval; Ref., Reference category; Multinomial logistic regression model.

Participants who had diabetes in both 1st and 2nd degree relatives had 6.28 increased odds of having retinopathy compared with participants who did not have a family history of diabetes. Diabetes in both 1st and 2nd degree relatives was associated with a higher duration of diabetes (years) and higher HbA1C (%).

Diabetes in 1st degree relatives was associated with higher HAM-D total scores.

### PHQ-9 depression symptoms prevalence.

**Figure 2:**
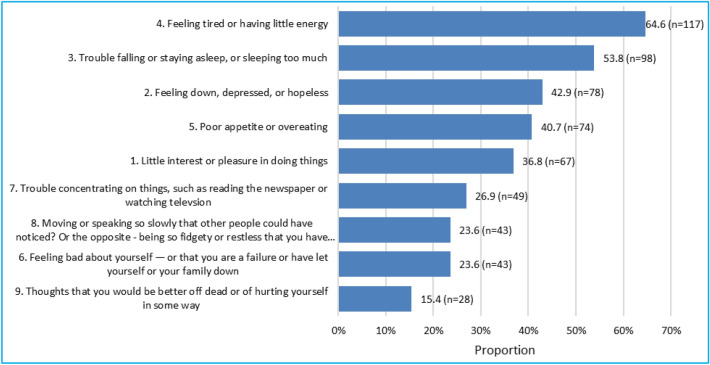
Prevalence of PHQ-9 aspects in descending order (N = 182).

Figure 2 depicts the prevalence of various depression symptoms measured by PHQ-9.

Most respondents had experienced profound fatigue or low energy levels, with over half indicating trouble with sleep patterns. Notably, a significant portion, comprising 15.40% of respondents, reported thoughts of being better off dead or of hurting themselves in some way.

### Diabetes type 2 regardless of family relation

**Figure 3:**
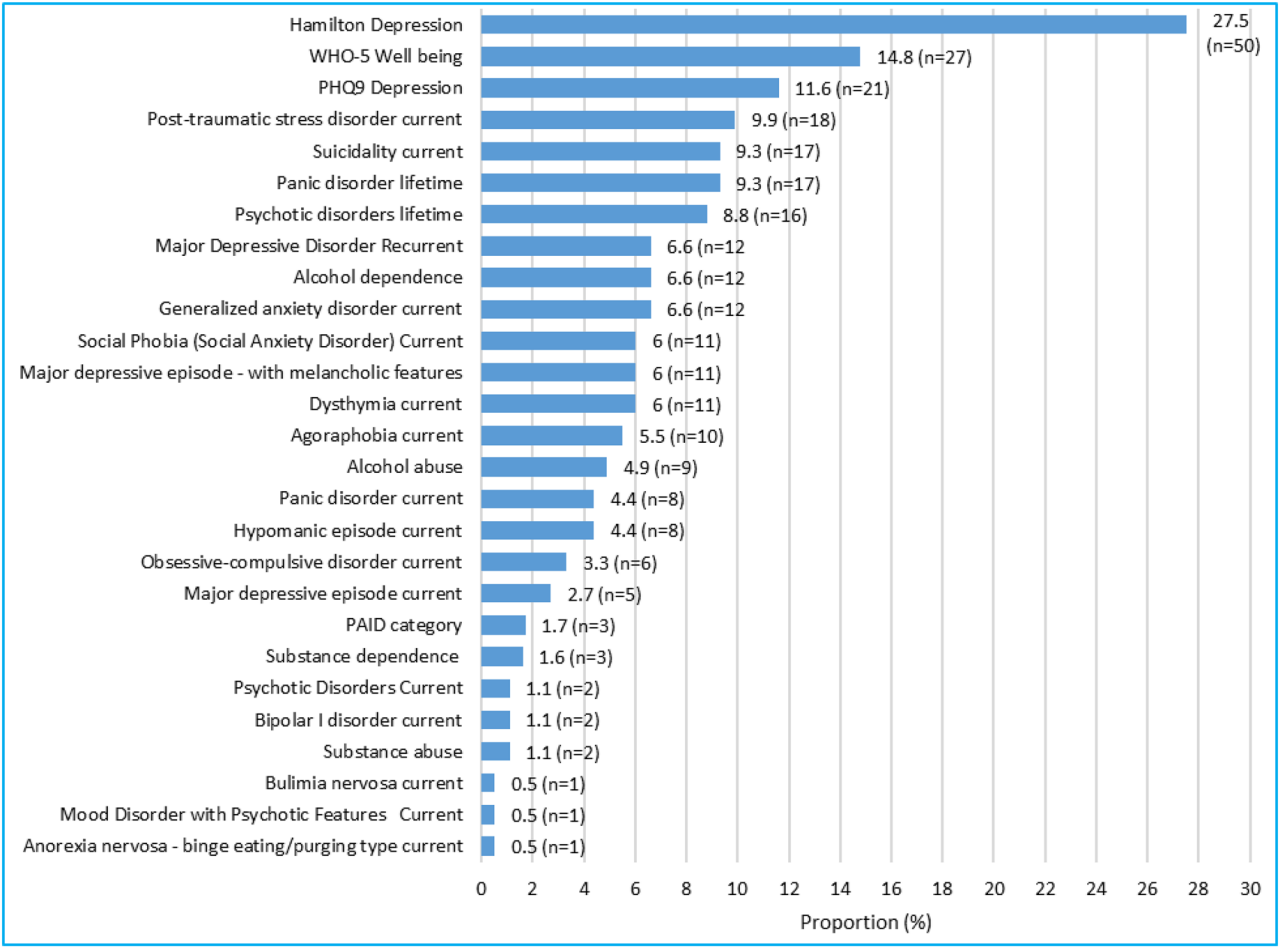
Prevalence of HAM-D, poor WHO wellbeing, PHQ-9, PAID and DSM-5 mental disorders in descending order (N = 182).

Table 5 summarizes the prevalence of the various aspects of mental health disorders as measured by the various instruments used in all the 182 patients attending the clinic, regardless of family relations. The prevalence of these various conditions is summarized in Fig. 3 in descending order. HAM-D was by far the most common mental health disorder while eating disorders (bulimia and anorexia) were the least with suicidality occupying the third position in the descending order, while elevated PAID was among the least.

**Table 5:** Prevalence of PHQ depression, poor WHO-5 wellbeing, PAID, HAM-D, emotional distress and DSM-IV mental disorder in Type II diabetes.

Variable	Category	Total (N = 182)
PHQ-9 depression	Not severe (< 10)	161 (88.5%)
	Severe (≥ 10)	21 (11.5%)
HAM-D depression	Not severe (< 7)	132 (72.5%)
	Severe (≥ 7)	50 (27.5%)
PAID	Low level (< 40)	176 (98.3%)
	Elevated (≥ 40)	3 (1.7%)
WHO-5 well being	Poor (< 13)	27 (14.8%)
	Good (≥ 13)	155 (85.2%)
Major depressive episode current	No	177 (97.3%)
	Yes	5 (2.7%)
Major depressive episode—with melancholic features current	No	171 (94.0%)
	Yes	11 (6.0%)
Dysthymia current	No	171 (94.0%)
	Yes	11 (6.0%)
Major depressive disorder recurrent	No	170 (93.4%)
	Yes	12 (6.6%)
Suicidality current	No	165 (90.7%)
	Yes	17 (9.3%)
Hypomanic episode current	No	174 (95.6%)
	Yes	8 (4.4%)
Bipolar I disorder current	No	180 (98.9%)
	Yes	2 (1.1%)
Panic disorder current	No	174 (95.6%)
	Yes	8 (4.4%)
Panic disorder lifetime	No	165 (90.7%)
	Yes	17 (9.3%)
Agoraphobia current	No	172 (94.5%)
	Yes	10 (5.5%)
Social phobia (social anxiety disorder) current	No	171 (94.0%)
	Yes	11 (6.0%)
Obsessive–compulsive disorder current	No	176 (96.7%)
	Yes	6 (3.3%)
Post-traumatic stress disorder current	No	164 (90.1%)
	Yes	18 (9.9%)
Alcohol dependence	No	170 (93.4%)
	Yes	12 (6.6%)
Alcohol abuse	No	173 (95.1%)
	Yes	9 (4.9%)
Substance dependence	No	179 (98.4%)
	Yes	3 (1.6%)
Substance abuse	No	180 (98.9%)
	Yes	2 (1.1%)
Psychotic disorders lifetime	No	166 (91.2%)
	Yes	16 (8.8%)
Psychotic disorders current	No	180 (98.9%)
	Yes	2 (1.1%)
Mood disorder with psychotic features current	No	181 (99.5%)
	Yes	1 (0.5%)
Bulimia nervosa current	No	181 (99.5%)
	Yes	1 (0.5%)
Anorexia nervosa—binge eating/purging type current	No	181 (99.5%)
	Yes	1 (0.5%)
Generalized anxiety disorder current	No	170 (93.4%)
	Yes	12 (6.6%)

*Row percentages.

^†^Fisher’s exact test; *p* value, Significance level.

Table 6 summarizes the independent predictors of depression in Diabetes. These predictors are diabetic foot problems, poor WHO-5 Wellbeing, and suicidality.

**Table 6:** Independent predictors of diabetes depression in T2DM.

Variable	Category	Adjusted odds ratio	95% Confidence interval		*p* value
			Lower	Upper	
Diabetic foot problems	No	Ref			
	Yes	1.59	1.24	2.04	** < 0.001**
WHO-5 well being	Poor (< 13)	Ref			
	Good (≥ 13)	0.59	0.41	0.83	**0.003**
Suicidality current	No	Ref			
	Yes	1.57	1.04	2.42	**0.043**

Significant values are in [bold].

Ref. = Reference category; *p* value = significance level; negative binomial model; Other variables in the model: PAID emotional distress, Major depressive episode—with melancholic features current, Major depressive disorder recurrent, Panic disorder current, Post-traumatic stress disorder current, Generalized anxiety disorder current.

## Discussion

### Preamble

This report serves two main purposes: to provide context-appropriate evidence for Kenya to support the holistic and liaison approach to the management of T2DM and secondly to contribute to the global data pool by offering recommendations that can be replicated in similar contexts.

To our knowledge, this is the first Kenyan cohort study that reports different genetic loading (family history in different degrees of relations) and the significant independent predictors of T2DM and the associations between T2DM and socio-demographic characteristics, physical conditions, and mental disorders. As far as we were able to establish, this is not just a first for Kenya but also in Africa.

### Family history

The finding of 45.1% of family history is lower than the reported 60–68.9% in the literature. This discrepancy could be attributed to the selection of the research participants in various studies. Ours was an outpatient clinic that excluded those admitted and presumably with severe forms of T2DM and possibly higher genetic loading. However, the finding of 45% is still significant for the Kenyan context, given that it is a non-modifiable contributor, hence the need for concerted efforts to focus on modifiable factors that are feasible in the Kenyan situation with limited resources, besides genetic counseling.

### Social-demographics

There were no significant differences between a family history of diabetes and all the socio-demographic variables studied, nor was any socio-demographic variable a predictor of T2DM. It is noteworthy that smoking status was not associated with any type of T2DM family history. This could be a reflection of no history of smoking in the cohort studied, a practice that should be encouraged and no doubt the policy in Kenya to put social pressure against smoking and also counseling at the clinic. Another unexpected finding though not reaching a significant level was that of only 25% males of the total clinic patients. This could be explained as a gender preference to attend this public facility or a reflection of the differential gender prevalence of diabetes in the communities served by this public facility. A further possible explanation is a trend though not significant that the overwhelming majority (84–87%) of females, as opposed to 12.5% -22.7% of males, had a family history of T2DM. Mixed methods studies are required to explain these findings.

### Physical conditions and biomarkers

Our study has shown that the higher the genetic loading the higher the association of retinopathy with T2DM in 1st and 2nd degree relatives compared with other levels of family history. Additionally, the highest association with diabetes in both 1st and 2nd degree relatives was found for the duration of diabetes in years, hypertension, and two specific biomarkers—HbAIC (%) and blood pressure (BP). BP and by extension hypertension can be easily monitored in the community, with the support of a relative, using easily available and affordable but reliable and valid BP monitors at home or the nearest health facility. This is an efficient way of monitoring and preventing T2DM, especially in those with a high genetic loading of diabetes. There is a new policy for every Kenyan family and all the individuals in that family to be reached at their homes on a regular basis by the newly created cadre of Primary Health promoters. They will not only attend to health promotion through awareness and attend to minor ailments but also take blood pressure. This community approach to monitoring blood pressure if successful is likely to have a critical impact on diabetes. Routine screening for blood pressure achieves extra significance given that 16.5% of our study patients were aged 60 + on age group. It is at this age group where various dementing conditions increase and hypertension is a risk factor for dementia^48,49^. The same principle applies to a routine determination of HbAIC (%) in those with the highest family loading of genetic risk for diabetes. In the Kenyan situation, blood samples for these can be taken at the nearest facility, and analysis carried out in that or the nearest available facility. Routine liaison consultation with the easily available ophthalmic clinical officers, (with the option to refer) for ophthalmoscopy is required for all patients with T2DM and more mandatory for patients with the highest genetic family history of T2DM in all diabetes clinics everywhere. Good history taking on the duration of diabetes is a routine practice that is reemphasized.

Even where there is no significant association with a family history of T2DM, our findings suggest there is a need for liaison practice, especially with renal and cardiology expertise. This expertise is usually but not always, available at all the 47 County Referral and Teaching hospitals in Kenya including the hospital where this study took place. While all physical conditions associated with T2DM were found in this cohort, only diabetic foot problems predicted depression. The holistic approach in that clinic could have mitigated other physical conditions as predictors of depression.

### Mental disorders

Although there was co-morbidity of diabetes with various mental disorders including alcohol abuse and dependence, WHO-5 wellbeing and diabetic stress, only depression, as determined by HAM-D was significant but less common in those with the highest level of genetic loading i.e., in both 1st and 2nd degree relatives.

Unlike HAM-D, PHQ-9 did not show any significant trends, suggesting the HAM_D scale is probably more sensitive and also the possibility that it is more valid than PHQ-9 in the type of patients we studied. While we do not have a conclusive explanation for this finding, we note that our sample size was small so no strong inferences could be made. Nevertheless, we venture a plausible explanation.

Firstly, if there are other family members with diabetes you are less likely to be depressed or anxious because there is support around you to help with your diabetes, therefore, less diabetes distress and more knowledge and understanding of diabetes.

However, we do not know whether individuals were living alone, an unlikely possibility in the Kenyan social-cultural context, if not they could still have family contacts through the still operational extended family and family social support systems in Kenya, though, this is diminishing towards nuclear centered families. It is also possible—that if there was a more laissez-faire attitude towards diabetes in relatives, then that might also lead to lower levels of anxiety and stress. On the other side, this attitude could at the same time lead to poorer glycemic control and so increased risk for microvascular disease. Either way, there are important implications for practice—screening for diabetes as well as depression, and improved knowledge of the risks of diabetes. The depression could be secondary to the onset of T2DM and most likely related to the burden of care in patients with T2DM.

The prevalence of various mental disorders found in this study was less than has been reported previously in the wider non-diabetic general clinical population in Kenya during a past study^50^.

Although there were no significant associations of all other types of mental disorders with a family history of T2DM, the high co-morbidity, ranging up to 13.6% and with a particular note of suicidality, calls for liaison with mental health experts in the management of T2DM. Apart from the findings on family relations, there are other incidental but clinically important findings. Of note is that although the association with psychotic conditions did not achieve significance, these psychotic conditions could negatively affect the overall management of T2DM. It is likely that the patients with these symptoms were treatment naive or not yet diagnosed and had therefore not received appropriate treatment for their psychosis. We therefore recommend routine screening for mental disorders using easily self-administered tests for all patients attending diabetes clinics. This self-screen is recommended because diabetologists are not necessarily experts in mental health and may not have the time to take a full history or make a diagnosis using a clinician-administered tool. Secondly, more importantly, the patients themselves may not be aware of, or may not feel able to report their mental health problems. Thirdly, joint management of diabetes and any mental disorder may have a better outcome for both conditions. This is feasible at local health center facilities, which are widely accessible at the community level, using stepwise upward referrals to the higher levels where there is the necessary expertise. Recommendations for treatment can then be provided using a stepwise downward referral process so that the patients can be managed in their communities. This will enhance the availability and accessibility of services and benefit capacity building in skills at the grassroots level.

The low-level prevalence of emotional stress (2.7%) does not allow us to test significant associations. While being diagnosed with diabetes can cause anxiety and depression and lead to emotional distress, the cause-effect could also be bi-directional—i.e. diagnosis leading to emotional distress or conversely emotional distress from other unidentified factors such as physical conditions leading to anxiety and distress. This calls for a qualitative approach that explores at a clinical level any directional relationship in a particular patient.

This finding of 2.7% prevalence of diabetes-related emotional distress is one of the lowest as compared to 12.8–46% reported in the literature^51–53^. We speculate that this is a reflection of the type of engagement of the patients that goes beyond the prescription of drugs in that particular clinic. It is the integrated management of diabetes that we speculate reduces emotional distress within a setting where the patients are fully educated on their conditions and management. It is likely that the levels of emotional distress would be similar to those reported in the literature for other situations and clinics that do not incorporate such holistic practices. If indeed that is the case, then it is a reflection of good practice in that specific clinic which could be replicated elsewhere.

Only combined methods—quantitative and qualitative have the potential to delineate these associations. Overall, our findings suggest the need for screening for depression, WHO-5 wellbeing, and suicidality in routine clinical management of T2DM at least in all patients with T2DM. Any positive screening findings should be integrated into the management of the patient.

## Conclusions

Family relationships are important in both physical disorders and depression, suggesting shared genetic predisposition, and/or modulation by shared environmental factors. Depression emerges as the common mental disorder in individuals with Type 2 Diabetes, irrespective of relational factors. Additionally, all examined patients exhibited various mental health concerns and DSM-5 disorders. This Kenyan study contributes to the global database on the topic of Types of diabetes and family relations and associated mental and physical conditions. We have achieved all our aims for this study.

Based on all the achieved general and specific aims, we have suggested some clinical and community health practices and policies.

### Limitations and recommendations to overcome the limitation

This study was carried out in a cohort of patients attending a diabetes clinic and therefore does not reflect the wider population of people with T2DM. This study excluded those untreated patients in the community or where clinics do not provide psychoeducation as in this clinic. Conversely, this holistic approach could be replicated in other clinics and contexts.

Secondly, we could not establish any directional relationships using the quantitative methods, given that our data is cross-sectional. Only mixed qualitative and quantitative methods could address this.

Although we achieved our aims, we recommend more studies at the community level to include those who may have T2DM and go for other services or are not treated by a specialist. Such a study though necessary for better understanding would be expensive and would require more complicated logistics.

## Data Availability

The datasets used and/or analyzed during the current study are available from the corresponding author upon reasonable request.
